# The effect of enhanced external counterpulsation on anxiety and depression in patients with paroxysmal atrial fibrillation: a randomized controlled trial

**DOI:** 10.3389/fcvm.2025.1548839

**Published:** 2025-03-21

**Authors:** Fengqin Yang, Yan Zhang, Yu Jiang, Qinfei Wei, Xiao Cheng, Jingwen Xiao, Geng Chen

**Affiliations:** ^1^ Department of Cardiovascular Medicine, Fuzhou First Hospital Affiliated with Fujian Medical University, Fuzhou, China; ^2^ Cardiovascular Disease Research Institute of Fuzhou City, Fuzhou, China; ^3^ Department of Nursing, Fuzhou First Hospital Affiliated with Fujian Medical University, Fuzhou, China

**Keywords:** counterpulsation, atrial fibrillation, anxiety, depression, randomized controlled trials

## Abstract

**Objective:**

This study aimed to investigate the effects of Enhanced External Counterpulsation (EECP) on anxiety and depression in patients with Paroxysmal Atrial Fibrillation (PAF).

**Methods:**

A cohort of 100 patients diagnosed with PAF at the Fuzhou First Hospital between January 2023 and June 2024 were enrolled in this study. Patients were randomly assigned to either the control group (*n* = 50) or the EECP group (*n* = 50) in this randomized controlled trial. The control group received conventional antiarrhythmic therapy with β-blockers or propafenone, and the EECP group underwent EECP therapy in conjunction with pharmacological treatment. The EECP therapy protocol consisted of 60-min sessions, conducted five times per week over a four-week period. Blood pressure, heart rate, cardiac ultrasound-related indexes, Self-rating Anxiety Scale (SAS), and Self-rating Depression Scale (SDS) scores were compared between the two groups before and after treatment.

**Results:**

After the intervention, the systolic blood pressure, diastolic blood pressure, LVEDD and LAD of the two groups decreased significantly compared to the pre-intervention period, the LVEF was higher than before intervention, and the systolic blood pressure, LVEF, LVEDD, and LAD of the EECP group were significantly better than those of the control group. Anxiety and depression indicators were lower in both groups than before the intervention, and scores in the EECP group were significantly lower than those in the control group, suggesting that EECP significantly improved anxiety and depression levels.

**Conclusion:**

EECP, as a non-invasive treatment, is effective in improving anxiety and depression in patients with PAF.

## Introduction

In recent years, the prevalence of atrial fibrillation (AF) has escalated in tandem with the aging global population and the increasing prevalence of cardiovascular diseases. Current statistics estimate the global AF prevalence at 37,574 million cases, with projections suggesting a potential increase of over 60% by 2050 (1). In China, the prevalence of AF among residents aged ≥35 years is 0.71% and increases significantly with age (2). Paroxysmal atrial fibrillation (PAF), characterized by episodes of atrial fibrillation lasting seven days or less, which can either resolve spontaneously or with pharmacological intervention, typically endures for less than 48 h and may recur, potentially progressing to persistent atrial fibrillation (PeAF). As the disease progresses, most patients with PAF experience symptoms such as palpitations, chest tightness, and dizziness, leading to a decrease in quality of life, and with this comes a series of emotional changes, with anxiety and depression being the most common (3).

Enhanced External Counterpulsation (EECP), recognized for its safety, efficacy, non-invasiveness, and non-pharmacological nature, has garnered increasing interest among scholars worldwide. This physiotherapeutic modality augments coronary blood flow and ameliorates myocardial ischemia by applying external compression to the chest during diastole, thereby positively impacting cardiovascular health. Specifically, EECP has been shown to enhance coronary artery blood flow during diastole, which in turn alleviates myocardial ischemia and benefits patients with cardiovascular diseases. A study has found that EECP can significantly improve myocardial ischemia symptoms in patients with coronary angina (4). Furthermore, a study has indicated that the combination of EECP with pharmacotherapy can reduce the left atrial internal diameter in patients with AF and decrease the recurrence rate of AF (5). In recent years, the application of EECP has gradually expanded, and its potential to improve the psychological state of patients has gradually received attention. Relevant studies have found that EECP improves anxiety and depression in patients with chronic insomnia and angina pectoris (6, 7).

Currently, cardiovascular diseases focus on the holistic treatment of cardiac rehabilitation, and the psychological health of PAF patients should not be neglected. This study is designed to explore the impact of EECP on the anxiety and depressive symptoms experienced by PAF patients, to contribute novel insights into the comprehensive treatment paradigm for this patient population.

## Materials and methods

### Study design and ethics

A total of 100 patients diagnosed with PAF at the Fuzhou First General Hospital Affiliated with Fujian Medical University between January 2023 and June 2024 were enrolled in this study. Participants were randomly assigned to either the control group or the EECP group using a random number table method, ensuring equal distribution with 50 patients in each group. The control group comprised 30 males and 20 females with a mean age of 67.56 ± 6.32 years, while the EECP group included 32 males and 18 females with a mean age of 68.52 ± 6.27 years.

Inclusion criteria: ① patients met the diagnostic criteria of paroxysmal atrial fibrillation in the 2019 AHA/ACC/HRS Guidelines for the Management of Patients with Atrial Fibrillation and their conditions were stable; ② patients and their family members were aware of the content of the study and voluntarily signed the informed consent form.

Exclusion criteria: ① patients with a bleeding tendency or coagulation dysfunction; ② those who have serious conscious dysfunction and difficulty communicating normally; ③ those who have serious liver, kidney, lung, and other organ lesions or malignant tumors.

This clinical trial was conducted in accordance with the ethical principles of the Declaration of Helsinki (2013 revision) and its subsequent amendments. This study was reviewed and approved by the Hospital Ethics Committee (Ethical Approval No.: 202208003).

### Collection of baseline information

Baseline evaluations comprised: (1) demographic profiles [age, sex, body mass index (BMI, calculated as weight in kilograms divided by height in meters squared)]; (2) course of AF and CHA2DS2-VASc score; (3) lifestyle behaviors (smoking history, drinking history); (4) clinical comorbidities (hypertension, diabetes, and coronary artery disease); (5) pharmacotherapeutic regimens (β-blockers, Class I/III antiarrhythmics, calcium channel blockers, aspirin, rivaroxaban, statins); and (6) renal function biomarkers (creatinine, uric acid, and urea nitrogen).

### Grouping

According to the 2023 ACC/AHA/ACCP/HRS guideline for the diagnosis and management of atrial fibrillation, patients in the control group received individualized conventional medications such as β-blockers (e.g., metoprolol extended-release tablets, 47.5 mg, once a day), propafenone (150 mg, three times a day), or rivaroxaban (15 mg, once a day). Patients with hypertension, diabetes mellitus, and other disorders were treated with appropriate medications.

The EECP group underwent EECP treatment in addition to the use of conventional medication.

### EECP

EECP therapy was administered using the Air-bag Type Sequential External Counter-pulsation Device (Chongqing PSK-Health Sci-Tech Development Co., Ltd., China; Model: P-ECP/TI). Treatment comprised 60-min daily sessions, 5 days/week, delivered over four consecutive weeks.

A counterpulsation pressure of 0.025–0.045 MPa was used during EECP therapy and the following principles were followed: ① adjusting the therapeutic pressure to maximize the diastolic surge wave during counterpulsation therapy; and ② selecting the minimum therapeutic pressure while maintaining the principle of the highest surge wave. Patient treatment compliance was greater than or equal to 90%, EECP treatment was supervised by a cardiac rehabilitator, and there were no adverse events or withdrawals during treatment.

### Observation indexes

Blood pressure and heart rate measurements, including systolic and diastolic blood pressure, were recorded for both the control and EECP groups before and after the treatment intervention using Patient Monitor (PHILIPS, GS20, Philips Goldcorp Shenzhen Industrial Co., Ltd.).

Cardiac ultrasound evaluations [recording of left ventricular ejection fraction (LVEF), left atrial diameter (LAD), and left ventricular end-diastolic diameter (LVEDD)] were performed within 1 week before the start of the 4-week treatment and 1 week after the end of the treatment, and were completed by two ultrasonographers certified by ultrasound exams, who were unaware of the subgroups. Intraobserver variability was <5%.

Furthermore, based on previously validated literature (8), the Self-rating Anxiety Scale (SAS) and Self-rating Depression Scale (SDS) were administered to both groups to quantitatively assess the levels of anxiety and depression before and after the treatment. The scale self-assessment process and results were assessed and reviewed by two nurses who were professionally trained and unaware of the subgroups. The Chinese version of the SAS and SDS have been validated in the Chinese population with Cronbach's alpha coefficients of 0.73 and 0.79, respectively (9).

### Statistical processing

Data analysis was conducted using SPSS 27.0. Continuous variables were expressed as the mean ± standard deviation (*x* ± *s*), the independent sample *t*-test was used for comparison between groups, and the pairs of samples *t*-test was used for comparison before and after treatment. Categorical data were presented as frequencies (*n*) and percentages (%), with group comparisons performed using the Chi-square (*χ*^2^) test. The difference was considered statistically significant at *P* < 0.05.

## Results

### Baseline profile status of patients with AF

There was no statistically significant difference in the baseline data of gender, age, smoking history, drinking history, family history of hypertension, prevalence of hypertension, diabetes mellitus, coronary heart disease, BMI, and course of AF between the EECP and control AF patients, indicating that the two balanced groups were comparable (*P* > 0.05, Table 1).

**Table 1:** Comparison of baseline characteristics of patients with AF.

Variable	EECP (*n* = 50)	Control (*n* = 5)	Statistic (*t*/*χ*^2^)	*P*
Age (years)	68.52 ± 6.27	67.56 ± 6.32	−0.763	0.448
Male	32 (64.0)	30 (60.0)	0.170	0.680
BMI (kg/cm^2^)	23.24 ± 2.05	22.82 ± 2.37	−0.381	0.704
Course of AF (years)	3.74 ± 1.50	3.62 ± 1.65	−0.944	0.347
CHA2DS2-VASc score	2.38 ± 1.26	2.26 ± 1.31	−0.468	0.641
Smoking history	18 (36.0)	14 (28.0)	0.735	0.391
Drinking history	25 (50.0)	29 (58.0)	0.644	0.422
Hypertension	29 (58.0)	34 (68.0)	1.073	0.300
Diabetes	22 (44.0)	19 (38.0)	0.372	0.542
Coronary artery disease	27 (54.0)	26 (52.0)	0.040	0.841
Degree of coronary artery lesions		4.460	0.108	
Single-vessel lesions	9 (34.6)	11 (40.7)		
Two-vessel lesions	15 (57.7)	9 (33.3)		
Three-vessel lesions	2 (7.7)	7 (25.9)		
PCI	20 (40.0)	22 (44.0)	0.164	0.685
Drug history				
β-blockers	38 (76.0)	36 (72.0)	0.208	0.648
Calcium channel blockers	24 (48.0)	21 (42.0)	0.364	0.546
Class I antiarrhythmics	16 (32.0)	15 (30.0)	0.047	0.829
Class III antiarrhythmics	12 (24.0)	13 (26.0)	0.053	0.817
Aspirin	28 (56.0)	26 (52.0)	0.161	0.688
Rivaroxaban	25 (50.0)	27 (54.0)	0.160	0.689
Statins	31 (62.0)	28 (56.0)	0.372	0.542
Renal function				
Creatinine (μmoI/L)	58.54 ± 8.94	61.24 ± 7.62	1.626	0.107
Uric acid (μmoI/L)	241.62 ± 13.45	246.04 ± 18.43	1.370	0.174
Urea Nitrogen (Mmoi/L)	5.49 ± 1.59	5.12 ± 1.36	−1.245	0.216

Data for categorical variables are expressed as absolute numbers (percentages) and continuous variables are expressed as mean ± standard deviation. CHA2DS2-VASc Score represents the atrial fibrillation stroke risk score. AF, atrial fibrillation; EECP, enhanced external counterpulsation; BMI, body mass index; PCI, percutaneous coronary intervention.

### EECP effects on blood pressure and heart rate

Before the implementation of the intervention, there was no statistically significant difference in the comparison of systolic blood pressure, diastolic blood pressure, and heart rate levels between the EECP and control groups (all *P* > 0.05). After the intervention, the systolic blood pressure (126.98 ± 12.39 vs. 139.12 ± 14.13, *P* < 0.05), the diastolic blood pressure (72.16 ± 11.92 vs. 78.28 ± 11.94, *P* < 0.05) and the level of heart rate (74.20 ± 9.51 vs. 76.38 ± 9.57, *P* < 0.05) of the EECP group and the systolic blood pressure (134.50 ± 12.56 vs. 140.70 ± 13.19, *P* < 0.05), the diastolic blood pressure (74.68 ± 10.79 vs. 77.64 ± 10.35, *P* < 0.05) of the control group decreased significantly compared to the pre-intervention period. While the systolic blood pressure indexes of the EECP group (72.16 ± 11.92) decreased significantly compared to that of the control group (74.68 ± 10.79, *P* < 0.05), and the difference between the diastolic blood pressure indexes and the level of heart rate was not statistically significant, although they were also reduced (*P* > 0.05, Table 2).

**Table 2:** Comparison of blood pressure and heart rate between the two groups.

group	*t*/*P*	*n*	SBP (mmHg)		DBP (mmHg)		HR (times/min)	
			Before	After	Before	After	Before	After
EECP		50	139.12 ± 14.13	126.98 ± 12.39*	78.28 ± 11.94	72.16 ± 11.92*	76.38 ± 9.57	74.20 ± 9.51*
Control		50	140.70 ± 13.19	134.50 ± 12.56*	77.64 ± 10.35	74.68 ± 10.79*	76.64 ± 9.84	76.36 ± 10.07
	*t*		0.578	3.014	−0.286	1.108	0.134	1.102
	*P*		0.565	0.003	0.775	0.271	0.894	0.273

Data for continuous variables are expressed as mean ± standard deviation. EECP, enhanced external counterpulsation; SBP, systolic blood pressure; DBP, diastolic blood pressure; HR, heart rate. Before and after the intervention.

*P < 0.05.

### EECP effects on cardiac ultrasound indices

Before the implementation of the intervention, there was no statistically significant difference in the comparison of LVEF, LVEDD, and LAD between the two groups (all *P* > 0.05). After the intervention, the LVEF of the EECP group (65.30 ± 4.98 vs. 46.34 ± 5.66) and the control group (52.62 ± 3.91 vs. 47.37 ± 5.35) were significantly higher and the LVEDD and LAD were significantly lower than before intervention, and the LVEF (65.30 ± 4.98 vs. 52.62 ± 3.91), LVEDD (44.69 ± 3.64 vs. 52.46 ± 2.66), and LAD (40.33 ± 2.08 vs. 41.24 ± 1.76) of the EECP group were significantly better than those of the control group (all *P* < 0.05, Table 3).

**Table 3:** Comparison of cardiac ultrasound indices between the two groups.

group	*t*/*P*	*n*	LVEF (%)		LVEDD (mm)		LAD (mm)	
			Before	After	Before	After	Before	After
EECP		50	46.34 ± 5.66	65.30 ± 4.98*	59.19 ± 5.83	44.69 ± 3.64*	42.30 ± 2.30	40.33 ± 2.08*
Control		50	47.37 ± 5.35	52.62 ± 3.91*	57.09 ± 5.25	52.46 ± 2.66*	42.45 ± 2.03	41.24 ± 1.76*
	*t*		0.940	−14.168	−1.891	12.174	0.350	2.363
	*P*		0.349	<0.001	0.062	<0.001	0.727	0.020

Data for continuous variables are expressed as mean ± standard deviation. EECP, enhanced external counterpulsation; LVEF, left ventricular ejection fraction; LVEDD, left ventricular end-diastolic diameter; LA, left atrial diameter. Before and after the intervention.

*P < 0.05.

### EECP effects on anxiety and depression

There was no statistically significant difference between the SAS, and SDS scale scores of the two groups before the implementation of the intervention (all *P* > 0.05). After the intervention, the above indexes of the two groups were lower than before the intervention (all *P* < 0.05). The mean SAS score after the intervention in the EECP group was 32.26 ± 3.30, significantly lower than 34.88 ± 4.20 in the control group, and the mean SDS score after the intervention in the EECP group was 34.32 ± 3.55, significantly lower than 37.70 ± 4.66 in the control group, which suggests that the EECP can significantly improve anxiety and depression (all *P* < 0.05, Table 4).

**Table 4:** Comparison of SAS and SDS between the two groups.

Group	*t*/*P*	*n*	SAS		*t*	*P*	SDS		*t*	*P*
			Before	After			Before	After		
EECP		50	34.74 ± 3.93	32.26 ± 3.30	6.944	<0.001	39.62 ± 5.46	34.32 ± 3.55	9.081	<0.001
Control		50	35.50 ± 4.59	34.88 ± 4.20	2.160	0.036	38.62 ± 4.85	37.70 ± 4.66	2.815	0.007
	*t*		0.889	3.467			−0.910	4.082		
	*P*		0.376	<0.001			0.365	<0.001		

Data for continuous variables are expressed as mean ± standard deviation. EECP, enhanced external counterpulsation; SAS, self-rating anxiety scale; SDS, self-rating depression scale.

## Discussion

The findings of this study revealed that, compared to the control group, the EECP group exhibited significant reductions in SAS and SDS scores, as well as decreases in systolic blood pressure. Concurrently, there was a notable increase in LVEF following four weeks of EECP treatment.

EECP has emerged as a widely utilized treatment modality for a spectrum of cardiovascular conditions, including coronary heart disease, heart failure, hypertension, and cerebrovascular disease, as well as diabetes mellitus (10–14). The therapeutic mechanism of EECP involves the application of an airbag device around the patient's lower limbs and buttocks. Under the guidance of electrocardiographic monitoring, the airbags are inflated sequentially, beginning in the diastolic phase of the cardiac cycle from the distal end of the extremities and progressing proximally. This sequence generates a retrograde pressure wave that propels blood in the arteries backward into the aorta, augmenting diastolic pressure within the aortic lumen, thereby enhancing myocardial perfusion and alleviating myocardial ischemia. During cardiac systole, as monitored, the airbags deflate, facilitating the emptying of peripheral arteries to accommodate the increased blood volume pumped by the heart. In terms of renal function, EECP has been shown to increase effective renal blood flow, suppress the renin-angiotensin-aldosterone system (RAAS), and decrease renin secretion (15). The pathogenesis of atrial fibrillation (AF) is multifaceted, involving inflammation, oxidative stress, autonomic dysfunction, cardiac structural remodeling, and myocardial fibrosis (16). It is established that AF can elevate RAAS activity, triggering a cascade of physiopathological responses (17). We hypothesize that the inhibition of RAAS by EECP may provide a theoretical basis for its potential impact on AF.

Some research results show that cardiac rehabilitation can improve patients' physical function, enhance their exercise capacity, and alleviate negative emotions, thus improving the quality of life and decreasing the death rate of cardiovascular disease by nearly 30% (18). Among them, EECP as a passive aerobic exercise has become the focus of current research in the field of cardiac rehabilitation (19). One study found that after 1 course of using EECP, the 6-minute walking distance (6MWD) of patients with refractory angina increased by 46 meters on average, and anxiety was significantly improved (20). The results of a prospective study in Denmark showed that after 12 months of EECP treatment, the adverse mood of patients with coronary artery disease was significantly improved, indicating that EECP can effectively treat anxiety and depression in patients with coronary artery disease, and some scholars believe that EECP can reduce the inflammatory factors in the blood of patients with coronary artery disease, and play the role of anti-anxiety and depression by alleviating the symptoms of angina pectoris in the patients (21). The results of this study showed that EECP was effective in improving anxiety and depression in patients with PAF, in which the decrease of SAS and SDS in the EECP group was significantly better than that in the control group. This is consistent with the findings of the above study that EECP improves negative mood in other diseases, but the pathophysiological mechanism is not clear. The results of a study by Yu et al. revealed that patients with PAF treated with EECP had a significantly lower rate of recurrence of AF at 1–3 months of follow-up compared with the control group (27.8% vs. 47.4%) (5). This may also be one of the reasons for improving anxiety and depression in AF patients to some extent.

In addition, this study found that EECP reduced blood pressure and heart rate in patients with PAF, and in particular, SBP decreased more significantly in the EECP group than in the control group. This is similar to the findings of Sardari et al. In their study, it was found that the resting systolic blood pressure of patients decreased from (125.59 ± 22.35) mmHg to (116.26 ± 14.93) mmHg after 35 sessions of EECP treatment (22). EECP reduces the afterload of the heart, which leads to an increase in cardiac output, thus reducing the EECP can also effectively reduce the secretion of antihypertensive substances and stimulate the secretion of antihypertensive substances, such as increasing the secretion and release of cardiac natriuretic hormone (CNS), increasing the secretion of prostacyclin (PGI-2), increasing the expression of nitric oxide (NO) and decreasing the expression of plasma endothelin-1 (ET-1), which can reduce the peripheral resistance and arterial pressure (23). This study also showed an increase in LVEF (65.30 ± 4.98 vs. 46.34 ± 5.66) and a decrease in LVEDD (44.69 ± 3.64 vs. 59.19 ± 5.83) and LAD (40.33 ± 2.08 vs. 42.30 ± 2.30) in PAF patients treated with EECP. A META analysis of EECP and heart failure also showed a beneficial effect of EECP on LVEF (SMD = 0.64, 95% CI: 0.29–1.00, *P* = 0.0004) (11). The results of another study of EECP in heart failure showed that EECP reduced LVEDD (63.02 ± 10.39vs. 49.23 ± 4.16) and increased LVEF (43.51 ± 2.24 vs. 65.21 ± 6.01) (24). All of the above studies confirmed the role of EECP in improving left ventricular systolic function, which is similar to the findings of the present study, but the improvement in LAD may be more important in patients with AF.

Due to the limitations of study design, ethical review, and practical clinical practice of EECP, this study did not have a placebo control group, which could have led to the observation of a placebo effect or improvement in the natural course of the disease. However, we used conventional standard therapy as a control, and significant changes in objective parameters (blood pressure, heart rate, and cardiac ultrasound parameters) still support the efficacy of EECP treatment and positively contributed to the improvement of patients' anxiety and depression. Many similar randomized controlled studies do not currently adopt a placebo design, probably considering the difficulties of clinical ethics and specific operations (12, 25). However, we still need to recognize the potential impact of the lack of placebo control, and future studies could further differentiate the specific effects through a three-arm design (EECP group, drug control group, and placebo group).

In this study, by randomly dividing PAF patients into conventional treatment groups and EECP group, it was found through comparison that EECP could reduce blood pressure and heart rate, improve cardiac ultrasound-related indexes, and effectively relieve patients' anxiety and depression. Through this study, we expect that EECP can provide a scientific basis for the mental health management of PAF patients, provide a reference for clinicians to formulate personalized treatment plans, and ultimately improve the overall prognosis of PAF patients. However, this study also has the following limitations. Firstly, the duration of the study was relatively short, and the sample size was not large enough to draw definitive conclusions. Secondly, there was a lack of placebo control. Finally, we did not conduct long-term follow-up to assess the long-term efficacy of EECP. Future research should address these limitations by using a three-arm design, employing larger sample sizes, and utilizing a large number of metrics to thoroughly investigate the long-term effects of EECP on mental health outcomes in PAF patients.

## Conclusion

This study found that EECP can effectively improve anxiety and depression in PAF patients. We believe that as the clinical application research of EECP continues to deepen, this non-invasive treatment will play a more important role in the comprehensive treatment of PAF patients.

## Data Availability

The original contributions presented in the study are included in the article/Supplementary Material, further inquiries can be directed to the corresponding author.
